# Situated anticipation

**DOI:** 10.1007/s11229-018-02013-8

**Published:** 2018-11-20

**Authors:** Ludger van Dijk, Erik Rietveld

**Affiliations:** 1 Department of Philosophy, Centre for Philosophical Psychology, University of Antwerp, Prinsstraat 13, 2000 Antwerp, Belgium; 2 Department of Psychiatry and Amsterdam Brain and Cognition, Amsterdam University Medical Center, University of Amsterdam, Amsterdam, The Netherlands; 3 Department of Philosophy/ILLC, University of Amsterdam, Amsterdam, The Netherlands; 4 Department of Philosophy, University of Twente, Enschede, The Netherlands

**Keywords:** Affordances, Anticipation, Ecological psychology, Enaction, Situated cognition, Architecture, Making, Skilled intentionality

## Abstract

In cognitive science, long-term anticipation, such as when planning to do something next year, is typically seen as a form of ‘higher’ cognition, requiring a different account than the more basic activities that can be understood in terms of responsiveness to ‘affordances,’ i.e. to possibilities for action. Starting from architects that anticipate the possibility to make an architectural installation over the course of many months, in this paper we develop a process-based account of affordances that includes long-term anticipation within its scope. We present a framework in which situations and their affordances unfold, and can be thought of as continuing a history of practices into a current situational activity. In this activity affordances invite skilled participants to act further. Via these invitations one situation develops into the other; an unfolding process that sets up the conditions for its own continuation. Central to our process account of affordances is the idea that engaged individuals can be responsive to the direction of the process to which their actions contribute. Anticipation, at any temporal scale, is then part and parcel of keeping attuned to the movement of the unfolding situations to which an individual contributes. We concretize our account by returning to the example of anticipation observed in architectural practice. This account of anticipation opens the door to considering a wide array of human activities traditionally characterized as ‘higher’ cognition in terms of engaging with affordances.

We live, as it were, upon the front edge of an advancing wave-crest, and our sense of determinate direction in falling forward is all we cover of the future of our paths. (James 1912, p. 36)

## Introduction

We know our way about an endless number of situations and adapt our behavior effortlessly. In everyday life we continuously adjust to the demands of the situation—keeping an appropriate distance to each other in public transport, changing one’s tone of voice when talking to a child, or interrupting someone in an appropriate way in a conversation. In doing so, we show responsiveness to the normative demands of specific situations as they unfold (Rietveld 2008). That is, our actions embody basic distinctions such as adequate or inadequate and better or worse in a concrete context.

When skilled, our situated adjustments can unfold quickly and often require little explicit deliberation or reflection. But skilled agents can act adequately and appropriately on larger timescales as well. For instance, when driving to a store, writing a text, or building a house, skilled individuals also adjust their activity in an anticipatory manner—people act adequately by anticipating situations as they unfold across larger scales, although often in a less certain manner than activities on smaller timescales. Nonetheless, on these larger timescale too, it seems we tend to act appropriately and our action, be it *reflective or unreflective*, is adjusted to fit the situation.

Situated activity unfolding across small timescales has been explicated in terms of an individual’s skilled responsiveness to the relevant opportunities for action (Rietveld 2008)—i.e. to relevant *affordances* (Gibson 1979, p. 127, see below). For instance, in the context of an architectural design process improving the height of a door can be a relevant affordance that invites the architect’s response. Likewise, as we will show, when watching skilled architects at work one can observe a responsiveness to affordances that unfold across much larger timescales, sometimes taking many months. When asked to create an architectural installation in a space familiar to them, skilled architects might for instance sense that they will be able to do so; they might agree to do the project and make a start or say that they envision what the installation could look like there. This is the kind of anticipation of large-scale possibilities for action, i.e. affordances, that we want to account for. It might seem counter-intuitive to extend the notion of affordances to include such large scales of activity. Indeed, affordances are often used in a restricted sense to apply only to dealing with the immediate surroundings here and now. From such a perspective, a distinction between “higher” and “lower” forms of activity seems inevitable, and *anticipating* the possibility to engage the former might seem to require a different account from the possibility to engage the latter. However, such a distinction between “higher” and “lower” forms of activity is just what our process-based account of affordances aims to avoid. Exactly how we should characterize such large scale possibilities for action and how this account then allows us to cash out anticipation in terms of responsiveness to such affordances will be the main questions answered in this paper.

To answer these questions, we have been participating with and observing architects at work. One of the present authors (ER) has been involved in this field for over a decade, being one of the founders of RAAAF, an experimental, multidisciplinary architecture studio in Amsterdam (see below). For the present study the other author (LD) has moreover been observing the architects and other skilled individuals at this practice for well over a year. This included a period of nine months in which the architects worked on making a new architectural art installation for RAAAF’s explorations of ‘The End of Sitting’. The End of Sitting is a long-term art-science research project that investigates the possibilities of breaking the habit of sitting by creating landscapes of affordances might in the future support living without chairs (Rietveld et al. 2015; Rietveld 2016). As the process of making an art installation takes a long time to unfold and consists in concrete activities, it is by being embedded in the practice of architects for an extended period of time that the details of anticipating such a process can be observed (Ingold 2013, 2018; Malafouris 2013; Mol 2002; Sutton 2008). The observations of (and participation in) these situated activities was guided by the question of how skilled architects are able to anticipate making an art installation that does not exist yet. The observations moreover offered us concrete real life examples of larger scale action possibilities in this practice of architecture.

To understand anticipatory responsiveness to large-scale affordances, we believe that we need a process-based account of affordances. To develop this we will start from the rich sociomaterial notion of affordances that we find in the works of Rietveld and colleagues (Rietveld 2008; Rietveld and Kiverstein 2014; Rietveld et al. 2018). Whereas in their work the spatial dimension of affordances was foregrounded, we will emphasize the *temporal* character of affordances that their approach implies in order to show how a process-based affordance approach can in principle *extend* its scope—so that it includes the domain of anticipatory activity unfolding over larger timescales too. By developing a process-based account of affordances in which affordances are determined in activity and intertwine across timescales we thus aim to provide a *scalable* notion of affordances that allows any aspect of human involvement, from quickly unfolding “simple” grasping, to “complex” architectural making unfolding over a larger timescale to be understood in equal terms.^1^

Even in their most intuitive rendering—as possibilities for action—affordances already imply anticipation (Van Dijk and Withagen 2016). That is, although they are available in the current environment, they pertain to that which the environment offers a skilled individual to do in the *future*. The ability to engage affordances then (i.e. to be invited by them, being responsive to them), is already anticipatory. Crucially, we shall argue below that the anticipatory aspect to affordances is retained at any scale at which affordances are organized in activity. The linchpin of our argument is that once affordances are seen as forming in process, they can unfold on the same temporal scale as the activities necessary to enact them. Our point will be that if affordances are tied to the unfolding of activities, then the anticipation that is found in engaging affordances in basic cases holds for any other scale as well. Architectural possibilities for action often take many months to unfold. The observed case of anticipating architects will offer a test to see how far we can take affordances once we develop a process account of them.

The basic structure of the argument will be as follows. In the next section (Sect. 2) we will briefly introduce the phenomenon for which we aim to account by presenting a real-life situation of anticipation in the practice of architecture. The following three sections (Sects. 3 to Sect. 5) will develop a process-view of Rietveld (2008) and Rietveld and Kiverstein’s (2014) account of affordances, for which we are indebted to Ingold’s (2011, 2013) considerations of the ‘process of making.’ First we introduce a temporal notion of activity (Sect. 3). We then introduce a notion of affordances (Sect. 4) in which an individual engaging with an affordance can be understood as continuing the practical engagements of communities of people that preceded him or her.

On the basis of this process-based notion of affordances, we can show how affordances, like activities, become nested and hang together. We show that new affordances continuously form on multiple timescales concurrently as they invite individuals to participate. This way, they set up the conditions for their own continuation, their development, growth and decay (Sect. 5). Having spelled out this process, finally, we will claim that like the smaller, basic, cases of affordances, large-scale affordances are anticipatory. By setting up the conditions for their own continuation these affordances have a direction of unfolding that individuals can grow responsive to (Sect. 6). They thus invite further participation and enactment. We will end by returning to our example of architectural practice and consider how our view accounts for the long-term anticipation we witnessed there.

## The Affordances of Making an Art Installation

As mentioned above we have been observing the architects at work, and these ethnographical observations will serve us as a test case for situated anticipation towards the end of this paper. The observed architects at RAAAF make site-specific work at the crossroads of architecture, visual art and philosophy. In our example^2^ the architects were asked by the Mondriaan Fund, a Dutch fund for the visual arts, whether they would be willing to create an art installation at the headquarters of the art fund in an old and monumental building in Amsterdam.

Neither this request nor the decision to take up the challenge was made *in vacuo*, but what is our main interest here is that the architects knew they could do it. In a sense, the architects saw the *affordance* of making an art installation, even though the art installation obviously did not yet exist. In fact, the senior architect would sometimes say that he “knew exactly what he wanted to do.” So how can we understand an architect clearly anticipating such a project as skilled responsiveness to affordances? How was the senior architect able to anticipate that his colleagues and he would be able to make an, not yet existing, art installation that would take over 9 months to design and make? It was this question on engagement with this large-scale affordance of making an art installation that guided the observations of the architects at work and it is this question that we aim to answer in the remainder of this paper.

**Fig. 1:**
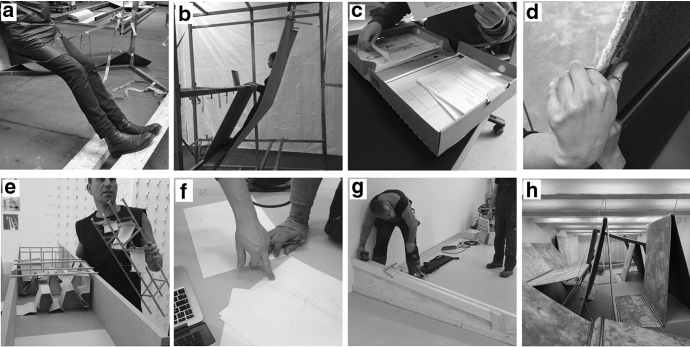
Six situations occurring as the making of an art installation unfolded. The aim of the art installation was to explore what a world without chairs could look like. **a** A tilted wooden plank being explored by the architects for its use in supporting the feet while standing. **b** Exploring a carpet for supporting the back and using to support a laptop to type. **c** Images, drawings and other paperwork being filed and checked. **d** Carpet being cut with a small pair of scissors. **e** Models that afforded comparing different design features. **f** Drawings on paper that specify the dimensions of a model. **g** A wooden beam being aligned to the room where the installation was being built. **h** The installation ‘Breaking Habits’ on the day of the opening, consisting of carpets suspended in the air by steel wires

Now, to get a feel for what such a ‘process of making’ involves (Ingold 2013), consider Fig. 1. Showing this we are not suggesting that the architects needed to plan the situations depicted in the figure all in advance when anticipating the making of an art installation. Rather, we merely wish to demonstrate the peculiar and diverse activities involved in enacting this affordance. As can be seen from the scenes shown in Fig. 1, the activities unfolding are often exploratory and involve practices that have not been well-trotted. Although the making of such an installation continues the practice of architecture, by its very nature as a work of art it also aims for novelty and not to simply repeat earlier works. The process of making an art installation is therefore a particularly interesting test case for studying anticipation.

The art installation that the architect decided to make aimed to show what a world without chairs could look like and needed to offer new ways of ‘supported standing’ to the visitors and employees of the art fund. It also needed to afford solitary working, comfortable waiting (it replaces the former waiting area), as well as afford having a meeting with multiple people. To the architects it was moreover important that the installation would be visually compelling and move earlier versions of their work into new territory. The practices the architects grew out of, their skills and the expertise they had gained from earlier collaborations on similar projects, together with the commission from the art fund offer them a significant opportunity for action: the affordance of making a new art installation started to invite participation.

We will return to this example of long-term anticipation towards the end of the paper. In the following sections we will first need to supply the details of a process-view on affordances. To make this theory as accessible as possible, we will interlace our view of affordances with examples from everyday life in- and outside of architectural practice. In Sect. 6 we will then reconnect our philosophy of affordances with the phenomenon of anticipating the large scale making of an art installation.

## Affordances in practice

To enable the concept of affordances to include new possibilities for action as these possibilities develop in human practices, Rietveld and Kiverstein (2014, p. 335) refined Chemero’s (2003, 2009) individual-based notion of affordances (as relations between abilities of an organism and features of the environment; see also Stoffregen 2003) by explicitly including the *practices* people can engage in. Affordances were defined as ‘relations between aspects of a material environment and abilities available in a form of life.’^3^ They thus emphasized that ‘affordance’ is an *open*-*ended* concept: affordances are open to include new possibilities as practices change. Crucially, by defining affordances as belonging to the form of life, i.e. patterns of behavior, ‘relatively stable and regular ways of doing things’ (ibid., p. 328), the affordances are tied to the practices people engage in, rather than to an individual’s ability. As we shall see below, although no longer defined relative to a particular individual, the chosen definition nonetheless allows affordances to be intimately related to the activities individuals develop.

In developing their notion of affordances, Rietveld and Kiverstein (2014) were chiefly concerned allowing the definition to be inclusive enough to allow the entire realm of human social significance to enter into it. The emphasis on the notion’s conceptual structure however may have distracted from the temporality that affordances entail (e.g. Van Dijk and Withagen 2016; Van Dijk and Rietveld 2017). In the following sections, we aim to further the development of Rietveld and Kiverstein’s relational view on affordances by explicating the *temporality* of the relata that they distinguish, how these relata relate *in process*, and how affordances can thus be seen as unfolding over time. Crucial for our process account of affordances is that we will understand concrete situations as *continuations* of real-life ongoing practices in terms of unfolding activities of individuals rather than as *realizations* of possibilities pre-existing *in abstracto*.

In the following, we aim to foreground the ongoing activity that is required for the continued persistence of affordances in the form of life. That is, we will ‘zoom in’ on the forefront of the unfolding forms of life (see Van Dijk and Rietveld 2017), where practices are being continued in particular ways by the activity of particular individuals. Thus, we gain sight of the affordances as they unfold in concrete situations. To do this, we will first discuss the notion of activity and then show how this plays a part in the concept of affordances. Subsequently we will ‘zoom out’ further and further to gain a view on the (manifold) activities that make up affordances at increasingly large timescales.

### Unfolding activity

**Fig. 2:**
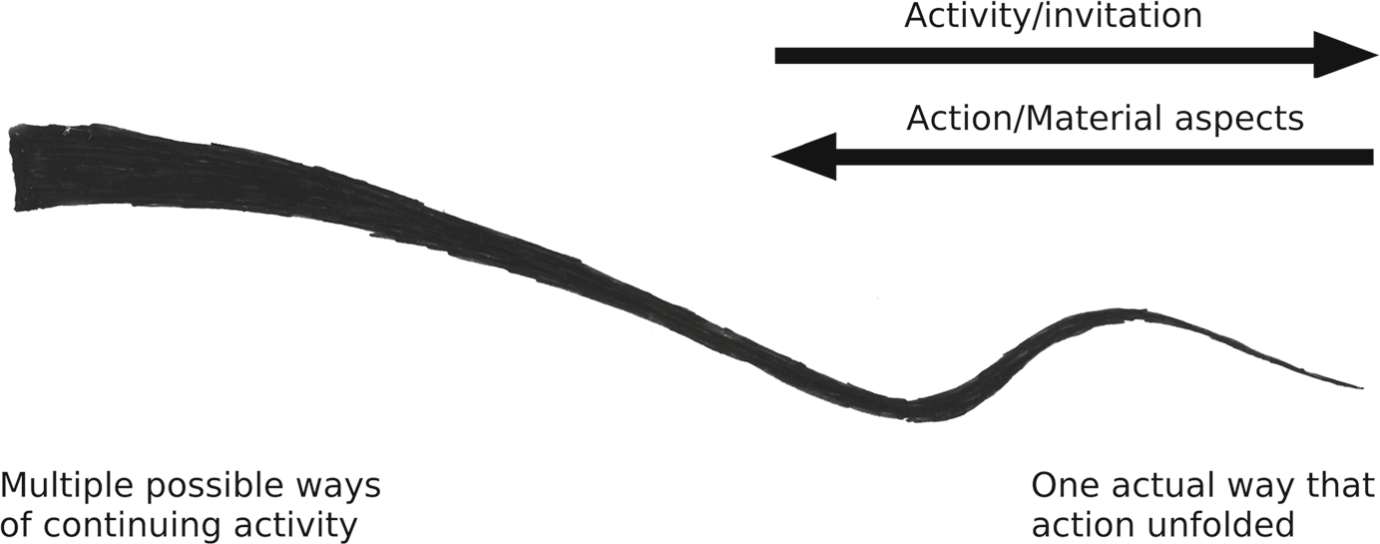
An activity as it unfolds into action. This view was inspired by Ingold (2011, p. 69, p. 84; 2013), Shotter (1983) and Schatzski (2012). As the line unfolds, it decreases in width to stress an activity’s increasing determinacy. From left to right, the multitude of possible ways in which materials can be coordinated and the activity can continue makes way for the one actual way in which material has been coordinated and the activity has unfolded. At the left-hand side, looking onward (i.e. to the right) the line indicates an activity, while starting from the right-hand side looking back on the line, it indicates a determined action and a certain way of coordinating materials (see Shotter 1983). We return to the material aspect and invitational character of the unfolding process (the arrows pointing right and left respectively) below in Sect. 4

Activities, whether performed by one individual or by many, unfold over time—they take effort and require skill (Gallagher 2005; Sutton 2008). This unfolding of activities highlights the fact that activities are a temporal process. This temporal process implies a past, a present and a future all at once (see Schatzki 2002, 2012; Van Dijk and Withagen 2016 for a fuller treatment). For example, if I am now painting my house, my history of skilled painting actions allows me to use a brush in a way that allows me to save my house from decay in the future. This temporal structure of the unfolding process is constitutive for the activity that it is. Stressing this, Schatzski (2012, p. 19 ff.) distinguished activities from actions, where an action is what the activity turns out to have accomplished (having painted a house, having written a text) and activity is the process of accomplishing the action (painting a house, writing a text). Notice that material aspects figure in such activities and that they are being coordinated in a certain way. The temporal relation between action and activity is depicted in Fig. 2.

The unfolding of activity stresses that it is ongoing and thus open to continuation. What action is performed can always be made more determinate. Consider the possibilities for action of a person’s fingers hitting the keys of a keyboard as he starts to type, for instance as an architect writes a text for a panel to hang on the wall next to an art installation. As his fingers get closer to the keys as he types, moving at a certain speed and direction, the number of keys that can still be hit decreases dynamically during the stroke. The number of local possibilities for action available (at his fingertips) decreases with his activity (and new possibilities for continuation also start to loom). In activity, possible ways of continuing make way for the actual way it has continued. What action is performed is therefore only completely determined when the activity, the performance, is over; when the key is hit, the word written and, to a lesser degree, the sentence or the text for the wall panel is finished (Shotter 1983).^4^ Equally, where and when materials figured in the activity, is also determined in the process. For instance, as part of an unfolding activity of sitting at a desk writing a text, in order to write a word a key was used for pressing down in the context of using other keys before and after it. Thus in activity a specific coordination of materials, a specific way of doing, unfolds. We will return to the role of materiality shortly. For now what is important is that conceptually, what action was performed and how materials entered into it, got determined in activity: time matters constitutively, and activity does not come *pre*-*formed*, but it is *performed*.

Importantly, any activity that appears as a finished *action* can be part of a continuing string of actions that forms a larger *activity* still unfolding. Like a key-press, activities are often nested in other activities (e.g. Reed 1996; Van Dijk and Rietveld 2017). The key press is nested in typing a word, which is nested in writing a sentence, and for example in writing a text for a wall panel.^5^ Note that these different scales involve increasingly many other activities (and other participants) contributing. The increasing determinacy thus unfolds across timescales concurrently and a new activity can re-constitute an action to be taken up in a larger functional unit (see Reed 1996). To give an example, in light of the writing of a wall panel (the unfolding whole), an aspect, like a word, sentence or paragraph may turn out to be ill-suited or misplaced. While determined in one sense, the action of writing such a sentence might thus take on new significance as the larger process unfolds (as an abandoned idea or as the inspiration to write an architectural manifesto). The larger process however unfolded the way it did, in part, because of the writing of the words and sentences that have since fallen from grace. In short, a future continuation of an activity can work backwards on past actions and change, to an extent, what activity it is that these past actions have been helping to accomplish (Dewey 1896; James 1912; see also Heft 1989; Noble 1981; Schatzski 2002; Shotter 1983; Van Dijk 2016; Van Dijk and Rietveld 2017).

The openness to continue differently however does not mean that activities are unconstrained. Their unfolding stresses the fact that activity has a *history* too: activities unfold within larger unfolding activities. These larger scale dynamics are constituted by many activities, performed by different individuals. These dynamics over longer timescales are the *practices* that make up the form of life and are continued into the present activity. Practices, the previously established regular ways of doing things, pave the ways in which activities on the smaller timescales are able to continue—that is, they are the (relevant) history *in terms of which* the current situation continues. Tap-dancing for instance has not been a regular way of doing things for architects in the past—it has not been part of their practices. Therefore, engaging in the practice of architecture will usually not invite tap-dancing. Doing so anyway would, save for exceptional circumstances, be a sign of mal-adaptation. It would be inappropriate and usually unsuccessful in continuing the practice of architects.

Acting along a practice thus constrains activities in a *temporal* sense. That is, it is the history of an already established practice in terms of which the current activity is unfolding that determines the possible ways of continuing (i.e. the width of the left-hand side of the line in Fig. 2). But as practices are ongoing, they too are increasingly determined and continued *in* and *by* the current activity. So, as James (in our opening quote) suggests, in writing a word, for example, the architect is ‘on the wave-crest’ of a sentence, which rides the wave of a whole manifesto and that of a developing view on, say, interventionist architecture. *Activities are always at the forefront of the practice, continuing it in situation*-*specific ways*: in terms of the past, but open to future possibilities (and so the practices of architects and tap-dancing might occasionally intermingle, for example when a particular person grows up in both—and his or her activities might one day turn out to have given shape to a new practice).

## Enacting affordances

So far we sketched a multi-scaled process of intermingling practices that unfold into the activities that continue it. Participating in these practices, we want to suggest, allows a skilled individual to *anticipate* the unfolding of the process. To make this claim about anticipation plausible, we will here add the concept of affordances to the process. This will serve two goals. First, as said, the concept of affordances is crucial to our account of *anticipation*. Our claim will be that the process that we described can be anticipated because it unfolds in terms of possibilities for action to engage with. Second, we have so far emphasized the ‘fluxy’ or ‘fluid’ nature of the process, so that it might seem as though we denied the persistence, regularity and stability within it.^6^ By highlighting the role of affordances within the process we hope to take away this concern.

When we discussed ‘activity’ and ‘action’ we highlighted their temporal relationship. As one and the same process, ‘activity’ characterized the process as it was still ongoing, while ‘action’ characterized the process once it was finished. Affordances—possibilities for action available in our form of life that form as material aspects and abilities intermingle—shed light on the same process but the notion (1) foregrounds the determination of the process achieved up to the current situation, and (2) points to the possibility to determine the process further.

Affordances in our view have the same temporal relationship that the action/activity dual has (Shotter 1983). As we will show in this section, the ‘backward-looking’ character of actions is allied by the role of ‘*materials*’ in the affordance concept. The notion of material aspects (of the environment) foregrounds which practices (i.e. which history of actions) have been established as the terms in which the situation is now available to continue (e.g. Costall 1997). The ‘forward-looking’ character of activities is captured by the ‘invitational’ character of affordances that are unfolding and foregrounds the open-endedness in ways to continue the current situation (for a discussion of this latter, experiential, aspect of an individual’s engagement with affordances see Van Dijk and Rietveld 2017).

Material constraints characterize the unfolding of the process in as far as it is finished and forms the given context for people. Recall how activity coordinates the way materials are used and thus gives new shape to them in writing a text, such as that of a wall panel that accompanies an art installation—coordinating chairs, key strokes, fingers, laptops and people—concurrently re-shaping the form that the panel is taking. The panel re-shapes with respect to the words, the sentences, and the lay-out, but also with respects to the text’s status as a series of bullet points, a draft emailed to others for feedback, a provisional text printed on paper to determine the layout, to the “final” text engraved into an aluminum wall panel. At any point, this multi-scaled process of writing then determines the terms in which the material aspects of the panel can be encountered: depending on the shape of the panel, it allows for particular possibilities for action. For example, once the text is engraved and glued to the wall changes would require far more effort (both socially and physically) than amending one of the earlier bullet points in an email. Materials thus ‘constrain’ the continuation of the process. That is, they characterize the determination that a practice, which continues into an unfolding affordance, has achieved up to this point.^7^ Material constraints on the behavior of people are thus very real and pose strong limits on the possibilities available – but they do not pre-exist our practices. They are continuous characteristics of unfolding practices: the previously established actions that have given shape to the possibilities currently encountered.

Together with an established past of constraining materiality, affordances also come with an openness to possibilities for future activity. Indeed, what has been determined so far are the ways in which one can go on; what possibilities for action can be performed in a given situation. Practices thus continue through the ‘inviting’ possibilities for action offered by the materials given. Note that material constraints/invitations and actions/activities have a similar temporal relation: affordances too are at once pertaining to a *past* of shared practices in which materials figured, while also *currently* affording, and affording a *future* continuation of activity. The temporal relation between materiality and activity is thus not one of succession—the one merely preceding the other (Gibson 1975; see Blattner 2005; Schatzki 2012; Van Dijk and Withagen 2016). Instead, both materiality and activity take shape together within the same ongoing process.

Crucially to get to a “scalable” notion of affordances that will allow anticipation at any timescale of skilled engagement, we here propose to think of materials/invitations on the one hand and actions/activities on the other hand as two sides of the same process of unfolding affordances. These two sides of the process of unfolding affordances are in our account always discerned in relation to one another. The relation between the two is quite straightforward: in affordances the unfolded actions (previously) established in a practice form the terms in which materials currently invite further activity to continue that practice in a particular way. Having acquired skill within a certain socio-cultural practice (for example architecture) allows one to be invited to perform the activity that will continue the practice, or put differently, to enact an action possibility. The invitational character of affordances as they unfold and the performative character of activity are equivalent ways to describe the continuation and determining of the process. Importantly this being invited by affordances is, as we eluded to from the onset, *anticipatory*. It anticipates the actions that are unfolding in doing.

We saw that in our process-view an activity can work back on previous actions to make it, in Dewey’s terms, continue ‘into it’ (Dewey 1896, p. 359).^8^ The action of writing a sentence, we showed, might take on new significance as the larger process of writing, in which the sentence figures, unfolds. The sentence might become ill-suited and having written it a mistake as the argument takes shape. Thus an activity can continue along a previous action and this action together with the following activity then makes up a larger scale activity that is also being enacted. Now that we have explicated how affordances and activities are two aspects of the same process, we can extend our argument for the scalability of affordances. What goes for actions goes for affordances as well: the enactment of one affordance and the enacting of the subsequently inviting affordance can together make up a larger scale affordance that may equally invite participation.

**Fig. 3:**
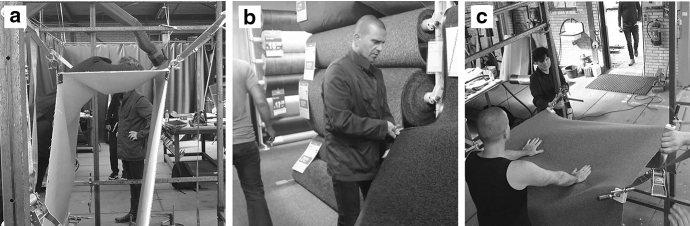
Three situations occurring during the exploration of different carpets. **a** A thin, single sheet of carpet suspended by lashing straps in a metal frame is being explored for the support it offers. Notice that the fabric is collapsing at the (left) fold as the person leans on it. **b** Rolls of dual-layered carpet being touched and compared by one of the architects in a store. **c** Back in the warehouse, a new sheet of carpet is suspended in the metal frame and the architect exerts force on it to explore how it holds up

Consider for example our architects who had at some point in the project explored the possibilities of various carpets in their experiments in the warehouse but found these fabrics to be too flexible and not retaining their shape so that the “position” that they tried to build did not work to support standing (see Fig. 3a). Dissatisfied with the carpets used for this position so far, and unable to work in the warehouse again the coming few weeks, the architects decided to go to a store immediately and look for stronger dual-layered carpet. If successful quickly enough they might have another go making a better position for the installation. The day’s work might then still end up advancing the overall process of making the installation. If unsuccessful the architects would have to wait for weeks before a new opportunity would arise to test fabrics. Sensitive to this last opportunity of that day to significantly advance the project, the architects thus set off swiftly to enact the affordance of making a better a position using a new fabric. Doing so involved being invited to look for nearby (wholesale) stores for carpet online, get into the van and take the right route to the store, seek the right fabric, change the old carpet in the warehouse for the new fabric, test it, and so on, all before the day was over.

**Fig. 4:**
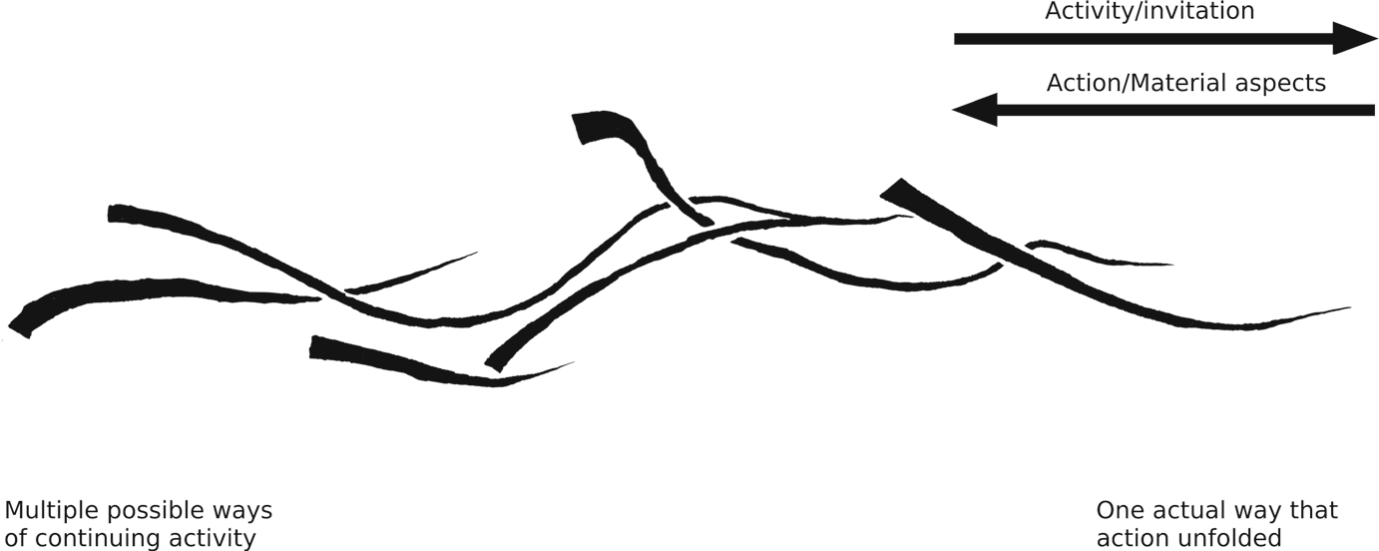
Small-scale actions/materials intertwining as they form a larger scale activity/affordance unfolding. The single larger scale activity can be viewed as a line similar to the six smaller lines that constitute its unfolding. Although in this figure the decrease in width is less pronounced than in Fig. 2, the larger scale activity does decrease in width, to stress the larger scale activity’s increasing determinacy. Again, from left to right, the multitude of possible ways in which materials and people can be coordinated with, and the activity can continue, makes way for the one actual way in which material has been coordinated and the activity has unfolded on this scale. At the left-hand side, looking onward, the lines indicate an activity—or an affordance inviting enactment, while starting from the right-hand side looking back on the line, the lines indicate materials having been used in action—an enacted affordance

This unfolding process of smaller scale invitations intertwining as they are enacted to form the larger-scale affordance of making a better position in the warehouse to which the architects responded is depicted schematically in Fig. 4. Note that, as they set out to go shopping, the architects were clearly responsive to the affordance of making a better position, indeterminate as it may be, and that their activities were coordinated such that the possibility could be enacted and become more determinate by doing so. That is to say, if they would have taken a wrong turn or spoken with the shop keepers too long, the possibility of making a better position that afternoon would have dissipated. Although requiring a fair bit of luck too, the architects’ intertwining activities together ended up reciprocally constituting the action possibility (affordance) of making a better position in the warehouse that day. The opportunity to make a better position structured a particular route, made some fabric rather than another invite buying, and paced conversations so that they did not take too long. Doing so in turn allowed the larger scale possibility—the affordance that the architects responded to when initially setting out to go to the carpet-store—to come closer to enactment.

Crucially, because affordances are often nested thusly, by means of acting someone can be concurrently enacting multiple affordances over larger timescales—bringing each to a different degree of determination as one goes on. For instance, the affordance to find the location of a carpet store online may have been quickly enacted and determined as the action performed, but this will not have determined the possibility to make a better position in the warehouse to the same extent (this affordance has been maintained as an inviting possibility, but might still fail). Acting such that affordances across timescales are increasingly co-determined is in fact what being invited by the affordance to make the position consists in. As we shall see in the next section, being responsive to the small scale allows the larger scale to keep inviting. Conversely, being simultaneously responsive to the larger scale, small scale activities are invited in terms of it.

## Setting up the conditions for its own continuation

In order to account for anticipation in terms of affordances, we have placed affordances in the process of continuing practices. We have been arguing that the affordances that have unfolded so far are the terms in which a process of ongoing practices can develop further—can now invite continuation. This involved the intermingling of both materials, which transform and persist over time, as well as of individuals acting as they encounter those materials as inviting activity. The process of affordances unfolding is thus an active process, one in which its constituents take form diachronically (Kirchhoff 2015; see Gallagher 2017). Crucially, we stressed that this process unfolds across multiple timescales concurrently, and that affordances at shorter timescales, like the actions that enact them, can intertwine and constitute another affordance unfolding across a large timescale (e.g. Fig. 4). Finally, we have suggested that affordances can continue a practice by inviting the activities that continue it. What we now want to add is that in doing so, *by inviting participation, the process sets up the conditions for its own continuation,* the conditions for its own maintenance and development. In this section we will spell out what this process entails and show how it requires anticipatory activity of its participants.

If materials and a person get enmeshed in activity as we have argued they do, then the role of the individual changes considerably. For the invited individual, the one that is partaking in the affordance as it unfolds—i.e. living along the line of Fig. 2—the unfolding in activity can have an experiential character (Rietveld 2008; Rietveld and Brouwers 2016; Van Dijk and Rietveld 2017). An individual that cares and is skilled enough to notice the affordance experiences the determining of activity—and thus the inviting character of the affordance being enacted—as having a ‘direction’ (Van Dijk and Rietveld 2017, p. 9; see James 1912, quoted above; Gallagher 2017; Heft 1989, 2001; Shotter 1983).

On short timescales, this direction along which affordances invite continuation is simply expressed as knowing what to do, as seeing how to continue—or as simply continuing it. For example when one is writing, reading back the first part of an unfinished sentence will often be enough to continue it satisfactorily. Likewise, when painting a beam, noticing that you missed a spot will immediately (and often unreflectively) draw out the appropriate response: having applied a touch of paint before you know it. An architect will immediately correct the height of a door that is too low and doing so reduces his directed discontent (Rietveld 2008; Wittgenstein 1967b). Crucially, what holds for such simple invitations enacted in the unfolding activity (Fig. 2) holds equally for the intertwinement of activities inviting participation (Fig. 4). The larger scale affordance will equally have a more or less determined direction for those invited to participate in it.

Larger scale affordances, like going to a carpet store to make a better position (e.g. Fig. 4) or building a house (or an art installation, see Sect. 6) have a direction of unfolding. Skilled individuals that have extended along a sufficiently long and relevant history of previous activities (i.e. they have a relevant ability), have acquired the responsiveness to attune to the direction of unfolding affordances along such larger timescales (Van Dijk and Withagen 2016). These “temporalized” individuals are therefore *able* to continue along these affordances—they can be invited by the larger timescale to participate and make it unfold further. Importantly, in the view developed here and in line with Rietveld’s (2008) account of unreflective expertise, only this skilled responsiveness or openness to the direction of unfolding is required to act along it. It is not necessary to know all the steps to be taken in advance, nor to have a ‘final state’ of the process specified in order to engage the possibility (nor, indeed, need there be such a state). Openness to the process to which the participants contribute as they act is all that is required of the skilled individual.

The (rather simplified) process depicted in Fig. 4 can be taken as a basic case. Suppose again that the figure represents the affordance to make a better position that supports standing. We have seen that enacting the activities of finding a carpet store and comparing fabrics once they have arrived there also enact—i.e. determine further—the unfolding of the affordance to improve the position, albeit to a lesser extent. Crucially, we have suggested that in doing so (once properly trained and skilled), one can be sensitive to both these unfolding scales simultaneously. Foregrounding the *larger scale* process now, we can see that participating in it will constrain the possibilities unfolding within (i.e. the width of the lines making up the larger scale)—the larger scale will invite locating a carpet store, rather than hanging around the warehouse any longer, to continue it. The small-scale activity of having found a store in turn keeps the affordance to make a better position open: getting into the van is now inviting, as is driving a little quicker to have sufficient time.

In this very basic case then, we find a larger scale process that can keep unfolding in as much as it invites its participant to enact the appropriate smaller scale affordances. We see a process of unfolding affordances, setting up the conditions for its own continuation. This skilled individual that is described as *anticipating* to make a better position is doing so in as much as he or she lets him- or herself participate in the unfolding of that affordance. This involves a responsiveness to the direction of the intertwining process. That is, responsiveness to how small scale activities allow the larger scale to keep inviting while the larger scale is forming the terms in which the small scales do so. If the individual were unresponsive (e.g. because other things are more important, because he or she is particularly unskilled at driving a van), the affordance to make a better position would dissipate, the activities diverge—it would not have invited the participants to enact it. Skilled participating thus requires being open to the moment that the process brings the materials out for what they afford—when the materials encountered invite the individual to determine them further.^9^

## Situated anticipating

Finally then, armed with these concepts and their temporal relationships, we can return to the phenomenon of long-term anticipating the making of the art installation we introduced in Sect. 2 and of which the above example of going to the store was an inviting part. In Sect. 2 we saw that skilled architects, upon being asked to make an art installation—an installation that will require many months of work by many different participants—are able to anticipate that they can do so. First off, we should note that there is a long history of architectural practices, maintained in academies, buildings, communities and studio’s, and continued in the path of activities of the individual architects (i.e. in their skills and abilities). Thus the abilities of architects have been developed, transformed and passed along to form a history of interactions up to the current situation in which the senior architect was asked whether he could design and make a site-specific architectural art installation.

As we highlighted above, the activities unfolding, although often almost banal (typing words, driving vans, painting wood), are at other times highly exploratory or involve practices that have not been well-trotted (standing on a tilted plank (Fig. 1a), grooming a carpet (Fig. 1d) that has been suspended by wires). Anticipating the making of an art installation thus has a much less stabilized unfolding than some of the everyday examples we used in the previous sections. Indeed, the ‘outcome’ of the affordance of making an art installation (e.g. Fig. 6h) is much less determined in advance than the affordance of, say, making a cup of coffee. But, we can now appreciate that it is *anticipated* by the participants in much the same way.

**Fig. 5:**
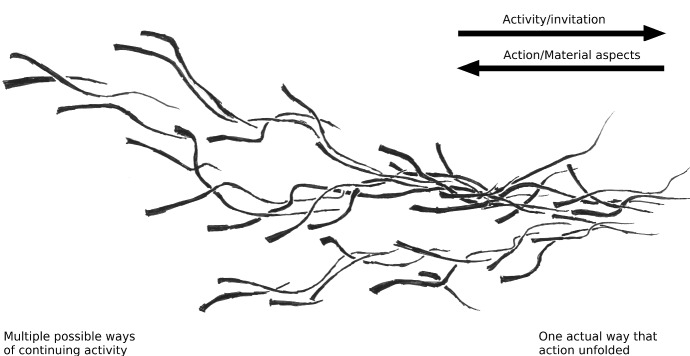
Small-scale actions/materials intertwining as they form a larger and larger scales activity/affordance unfolding. In this sketch three scales of activity can be discerned: single lines intertwine to form a strand of activity (such as the one depicted in Fig. 4), such strands in turn intertwine to form a larger scale unfolding process—consisting of the figure as a whole. At all these scales, the process is constrained by its own history, represented by their decreasing width. The number of scales depicted is however chosen for ease of presentation (indeed, this figure can be taken as the process visible when “zooming out” to the temporal context of the activity depictured in Fig. 2, but also as the process seen when “zooming in” on that figure further). From left to right, the multitude of possible ways in which materials and people can be coordinated with, and the activity can continue, makes way for the one actual way in which material has been coordinated and the activity has unfolded on this scale. At the left-hand side, looking onward, the lines indicate an activity—or an affordance inviting enactment, while starting from the right-hand side looking back on the line, the lines indicate materials having been used in action—an enacted affordance. In this section it will become clear that the activities within this process need not be performed by the same individual

Given the history of activities the architects have been developing, in which they have participated in making architectural works many times, making an art installation is an inviting affordance to them. Although a larger scale unfolding process, the making of the art installation, has the same structure as those of Figs. 2 and 4, but now consisting of many more intertwining activities yet again (see Fig. 5). It is an unfolding affordance, and as such it has a past of relevant practices (that we just briefly described), in terms of which a currently affording environment (e.g. Fig. 1a–h) invites continuing it into the future. In Fig. 5 the process of making an art installation is shown schematically.

**Fig. 6:**
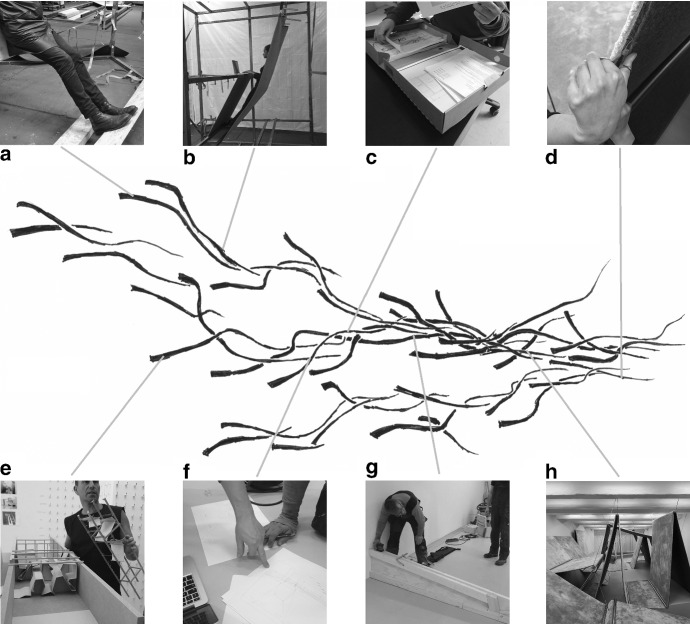
Actions/affordances intertwining as they unfold into the large-scale activity of making an architectural installation. The unfolding situations introduced in Fig. 1 have now been added to the figure to indicate their relation to the larger-scale process. The content of the photographs **a**–**h** are discussed in the text. Notice that the selected situations **a**–**h** are themselves part of situations consisting of unfolding actions/affordances that could be unpacked in same manner, resulting in a figure similar to Figs. 4 or 5 yet again

In Fig. 6 below we have added to the scheme of Fig. 5 the situations of unfolding activities that we introduced in Sect. 2 (but one can also see the situation depicted in Fig. 4 as one of its strands). These different situations can offer a variety in affordances if we take them out of the practical and historical context of the larger scale unfolding possibility of making an art installation (or to individuals with different histories of skill). The built model (Fig. 6e) may for example afford holding, preserving, collecting, or burning; the paper (Fig. 6c, f) may afford folding it into an airplane or writing on, and the wooden beam and the floor (Fig. 6h) may afford a balancing act or a tap-dancing routine respectively. But as part of the large-scale unfolding process of making an art installation, these materials invite very specific people to contribute very specific activities which in turn continue the process of making. Together these inviting affordances can be seen to set up the conditions for continuing from one into the other, thus forming the larger-scale process.

Looking at Fig. 6, early in the process (even before the commissioning by the art fund was known of) many materials invited being explored by the architects such as wood for supporting the feet while standing (Fig. 6a) and suspended carpet for supporting the back and using it to support a laptop to type (Fig. 6b). As the project started, models were built that invited comparison for different design features, such as the effect of lighting and the possibility to suspend carpet in the air on site (Fig. 6e). Situations developed in which images, drawings and other paperwork that had invited storing, could now invite to be checked again (Fig. 6c), but life-size models such as Fig. 6b also invited measurement, which invited making a measured drawing on paper (Fig. 6f) which in turn drew a caring and skilled carpenter into the process. By the drawing on paper he was invited to make wooden beams, which afforded measuring in the context of aligning it with the room where the installation was being built (Fig. 6g). As the process of making the installation, consisting of carpets suspended in the air by steel wires, neared completion (the installation determined as built) (Fig. 6h), to the architects it invited little more but grooming it. The carpet’s edge for instance invited to be cut with a small pair of scissors (Fig. 6d). But of course the installation now also started to invite supported standing to the people working at the art fund.

Considering the process of making the installation we find a large scale affordance unfolding. There is again a directionality and increasing determinacy—possible ways of continuing make way for the actual way it has been continued. In Figs. 5 and 6 looking back on the completed process of unfolding, we see the one actual way in which materials have been coordinated and the large-scale activity has unfolded along with the roles each of the smaller scale activities played in it. Looking forward (from the left of the figure), still in the ongoing process, we see a multitude of possible ways in which a diversity of materials can be coordinated and the activity can continue.

As a highly skilled architect, having dealt with design processes before, when first asked by the Mondriaan Fund to create an ‘End of Sitting’ style art installation for their waiting room the senior architect of this project immediately knew he would be able to do it. He could anticipate that they would be able to make a good installation. To him, the possibility of making an installation was simply an affordance available, one of which the enactment could be anticipated, given the history of earlier artworks realized, finding the right materials, and having caring and skilled people working with him. Although a by-stander might not have been able to anticipate the direction that the process is taking, the architects and the carpenter that have been deeply involved from the start certainly can. The more such participants are invited by the process to contribute their skills to the process, the more direction the process can take, and the more its participants will be able to attune to the direction of its large-scale unfolding (see also Noë 2012, e.g. p. 25 ff.), and thus, the more they will *anticipate* what needs to be done.

The process invites participants to intertwine with it and contribute their skills. They are invited to act and thus coordinate materials and transform them, so that these organized materials afford new activity to continue the process, the making of the installation. In short, the architects and other skilled individuals, familiar with architectural practices, can be invited to contribute their skills. By doing so, the larger scale process sets up the conditions for its own continuation—it forms the terms in which materials invite activity, from writing a sentence for a wall panel to seeing the opportunity to go to a store to buy carpet. As the large-scale affordance (the new installation as a whole) thus slowly nears enactment, the range of invitations for the architects grows smaller and may become very specialized and only inviting to a very few responsive participants (e.g. the ‘grooming’ in Fig. 6d). By that time, anticipating the large-scale project has long made way for the affordance of looking back on it. For others, participation has however just started, as the installation invites supported standing to the people working at the art fund, invites to be shown to visitors and, like the house we discussed, to be maintained and cared for in order to keep unfolding.

In this way a large-scale unfolding action possibility such as the making of an architectural installation can be anticipated. In fact, the process of making was anticipated both from the start of the project and along the way. However in our story, the attunement to the unfolding situation does not have an ‘object’ to which it refers. Neither is this attunement dealing with some ‘absent’ end-state, because it merely requires the openness and receptivity to the movement of an increasingly determining situation, seeing *along* the direction in which the situation is unfolding (Ingold 2013). Indeed, to us the phenomena of ‘having an image before one’s mind’, or of ‘knowing exactly what a design should come to look like’ offer a case in point. In our conceptualization, such expressions, sometimes used in situations of anticipating, are not a matter of literally having an image or any other mental content in one’s head. Rather, this phenomenon is an aspect of the skilled and attuned individual taken up in an unfolding and determining process.

As a final thought, the expression that one has the image before one’s mind then can be thought to expresses an openness, an attunement to, the large-scale unfolding affordance that one is contributing to—this goes equally for the design of an architectural installation, the writing of this paper or, say, the renovation of an old house. It should not be taken as *prima facie* evidence for a detached, isolated, individual mind, but as evidence for the fundamentally situated and relational constitution of the active individual. Indeed, trying to express the ‘image’, by drawing on a piece of paper, by writing or sketching, contributes to the process, which is determined further yet again. Thus, these explicating activities, themselves invited by the pieces of paper and people encountered in the unfolding process, are not merely *about* the process, but they are *of* the process—enabling it to continue by contributing new affordances ready to be enacted (Ingold 2011; Dewey 1958; James 1912; Van Dijk 2016).

## Concluding remarks

By foregrounding the unfolding of affordances we have shown by example that the concept of affordances can have a large enough scope and scale to deal with ‘higher’ cognition, such as cases of long-term anticipation and designing objects that do not yet exist. Affordances typically nest into larger scale affordances. Taking a process perspective, we have shed light on anticipation by highlighting the temporality of the concept of affordances: affordances turned out to have a similar unfolding structure to activity, including a past of determined structural and material features, a present situation that invites activity, and a future to be determined further—all at once. By inviting participation of people, affordances can weave together to form yet larger scale unfolding affordances. Setting up the condition for their own continuation, the unfolding structure of affordances returned at any scale of the process we considered. We saw it when considering basic activities, such as the affordance of getting into a van, when considering the larger-scale activity of making a new position by buying better suited carpet, and when considering the large unfoldings of an architectural art installation over the course of many months. Thus, in principle, affordances can extend along larger scales.

Affordances have a direction of unfolding that individuals can be responsive to if they are skilled, or that they can grow responsive to as they learn the relevant skills. From the process-based affordance perspective we have provided, such responsiveness or openness to the direction of unfolding is all that is required for anticipating future possibilities for action. That is, *anticipation in this view is part and parcel of engaging with affordances—at any scale*. Indeed, we normally are engaging multiple affordances simultaneously across timescales (Rietveld 2012). Any distinction between anticipation of large-scale affordances and the responsiveness to small scale inviting affordances is one of degree, not of kind.

We have aimed to foreground the richness of the temporal processes available to coordinate to. This however backgrounded the complexity of the job for the individual. Indeed, the individuals participating have only been implicitly represented in Fig. 5: they can be traced by following the activities in which they participate. An individual is usually caught up in multiple affordances, unfolding across multiple timescales simultaneously. What is required of the person is therefore not only an openness, but a *selective* openness (Reed 1996; Rietveld and Kiverstein 2014). An important ingredient of accounting for this lays in the neural (and other bodily) dynamics that have developed out of a history of past interactions, and now helps to shape the bodily readiness to act in one way rather than another (Bruineberg and Rietveld 2014; Bruineberg et al. 2016). It is beyond the scope of this paper to delve into the details of this hypothesis. Note however, that modeling the neural dynamics of an individual in terms of bodily readiness need not be understood as a representational capacity of that individual (Hutto and Myin 2013).

In a similar vein, while highlighting materiality we might have underemphasized the “physical” structures that are involved in the process. Indeed, physical structure is also only implicitly represented in the figures—and it too can be found by tracing along the activities in which such structure persists or transforms as it is taken up. This allows us to account for the many situations where “physical structure” plays a role in activity (e.g. taking measurements, knocking on a tabletop) without assuming it underlies all activity (Mol 2002; see Van Dijk and Withagen 2014, 2016). Note that such situating of physical structure is not meant to belittle the constraining of *materials*. We have taken great care to show through our examples how strongly possibilities for action rely on materiality-in-context (the use of a key in typing, of putting on a coat to catch a train, or of a cardboard model in making an architectural installation). Indeed, the constraining of materiality during unfolding activities was a crucial element for our approach: it pointed to the history of practices that determine the terms of which the current situation can be engaged with.

Using a process-based notion of affordances we have aimed to show how far we can take affordances in understanding forms of ‘higher’ cognition, including anticipating and designing a not yet existing architectural art installation. It would be interesting to push this line of reasoning further, and study the concrete details of activities that we tend to think about in abstract terms. For example, to develop this account of affordances further and apply it to cases of imagining and counterfactual reasoning. Because the field of situated cognition prioritizes the details of our concrete engagement in explaining cognition, we believe the field requires combining philosophical analysis with prolonged ethnographical study that scrutinizes actual practice (Mol 2002; Sutton 2008). We are currently working on detailing accounts of situated imagining and counterfactual reasoning by zooming in further on other aspects of our ethnographical observations. In so doing, we also hope to resolve one of the limitations of the current paper, which is that we did not consider failing actions and non-skilled or disinterested participation—cases where the process diverges or might lack in invitational character and some possibilities might even dissipate.

On the basis of our open-ended notion of affordances however, we believe that we will be able to account for a large diversity of concrete situations, and that even diverging situations might offer interesting and sometimes surprising opportunities for further action. In the end, we hope to show that in actual practice, situations of long-term anticipation or abstraction do not require less but more involvement, across broader, longer scales and more specialized activities; simultaneously engaging affordances on multiple timescales. Making this argument will however require us to observe and scrutinize the activities situated in actual real life practice much further. Doing so, we believe we will be able to put much of our human engagement, including our own philosophical reasoning, back into the unfolding processes out of which it flows.

## Footnotes

1 We should note from the onset that we do not wish to imply that such scaling is without limits. Indeed, what affordances are available will depend on the skills of people and, though open to change and development, these skills are always limited. More specifically, as we will see in Sect. 6, in any situation the unfolding affordances are diachronically constituted (Kirchhoff 2015; Gallagher 2017) by the activities that skilled individuals bring to bear. In an ongoing process of making an architectural art installation, these affordances can be anticipated by those individuals in as much as they can participate, i.e. to the extent that their activity can shape the unfolding process.

2 This example is taken from our ethnographical field work. The example is a simplified one because of the limited space we have here. More detailed observations of the activities will be presented in future work. All participants gave informed consent prior to observations and agreed to the publication of the photographs.

3 From their article (Rietveld and Kiverstein 2014) and subsequent work it is clear that the material environment in this account should be understood as a sociomaterial environment. See Van Dijk and Rietveld (2017) for details on this account of sociomateriality. For reasons of simplicity we will stick to the notion of material or materiality in our account of anticipation here. It is crucial to the human form of life that in action, materials and people are enmeshed and that materiality in the human form of life is sociomateriality (as will be seen in Fig. 5).

4 Note that we do not mean to imply that the future effect cannot be anticipated—we will in fact argue below for a way of engaging with the world that does justice to the lived experience of a skilled individual that anticipates the future because he or she is actively participating in it and can thus become sensitive to the way a situation invites him or her to continue it.

5 It is important to note that this nesting can be partial, fragmented and is not meant to imply any hierarchy. Activities (and, as we shall later see, affordances) often intertwine in a non-hierarchical manner: larger scales, activities that take longer, might cross paths and intermingle with the shorter ones and vice versa. Assigning a priority to any scale of enactment within the process of making a wall-panel for example would be a choice for analytical convenience (see Ingold 2011, p. 147; Bruineberg and Rietveld 2014, footnotes 2 and 3; Van Dijk and Withagen 2014).

6 A criticism raised at early process approaches to affordances (e.g. Noble 1981; Shotter 1983) was that it was unable to account for the determination that affordances, once understood in the context of a constellation of practices, already have (Costall 1997): “Potentially, the affordances of any object are entirely ‘open’” (Costall 1997, p. 77), nonetheless “things do not come to life as meaningful objects solely to the extent that *I* happen to draw them into my own activities” (Costall 1997, p. 78). Agreeing with this analysis, our approach aims to capture both the determination that a (shared) history of practices gives, as well as the indeterminacy that allows an individual the freedom to continue a practice in his or her own way, or to diverge from it.

7 In this view the determination of the affordances unfolded so far forms the materials as the materials they are up to this point. The local environment is therefore always already determined as having material structure to an important extent—namely to the extent that it can now invite an individual to contribute to unfolding the affordance further. This experienced constraining of materiality is crucial for our approach. The fact that on reflection material aspects of the environment appear to be pre-formed relative to the activity unfolding shows the increasing determination in activity. To use a gesture philosophers sometimes make in conversation: when knocking on a tabletop the brute reality of the physical structure seems undeniable. This shows how immersed one can be in a practice. The materiality of the table has been determined to such a degree that it no longer invites alternative ways of seeing it—it *must* be like this (Wittgenstein 1953 §101 ff.; §115; Wittgenstein 1967a, §349; see Van Dijk and Withagen 2014). Our account however aims not only to account for the properties singled out in knocking on a table, but includes the situation, following from a practice, that affords a philosopher the activity of singling out these properties. The possibility to use the table as an example is in fact one of the practices available in our form of life that give the table its brute reality each time anew (i.e. it is a ‘grammatical’ remark, explaining how the concept of ‘material’ works in some situations). We account for that, but we also want to account for the possibility that might develop in which a table’s persistence gives way: when a situation develops in which the table affords to be sawn in two, when its wood affords to be analyzed for ^14^C-isotopes or when only the mundane affordance to put a pint of beer on it invites.

8 Historically, Dewey’s discussion of a child burning its finger on a flame (in turn taken from James) serves to highlight how an action (and materiality for that matter) can take on further significance as it contributes to continuing a larger-scale activity (see Dewey 1896; Heft 1989; Shotter 1983). Dewey criticized the notion of the “reflex arc,” where a flame is the first stimulus, responded to by reaching for it. The next stimulus of burning is in turn followed by responding with a withdrawal of the hand. Crucially to Dewey, the flame is not first a (ready-made) stimulus and the withdrawal a response to that. Rather the flame and reaching, the burn and withdrawal can only be distinguished as stimuli or as responses when the entire event has unfolded. The burning of a finger is not a new experience added to the first seeing of a flame by mental association afterwards, but the burn develops the ongoing experience of a flame further: the withdrawal “is not *to* the stimulus; it is *into* it” (Dewey 1896, p. 359). The burn develops the act of seeing, namely into the “seeing-of-a-light-that-means-pain-when-contact-occurs” (Dewey 1896, p. 360; see Heft 1989). The flame then “is only completely specified as the stimulus it is when the response to which it has been the stimulus is complete” (Shotter 1983, p. 27).

9 This is characteristic of the ancient Greek notion of ‘kairos,’ the moment that must be seized when everything in the unfolding process is in alignment (see Smith 1969; Ingold 2011, p. 54). Skilled individuals participating in a process do seize this moment. Being in alignment should however not be confused with materiality being fully determinate. Participating in the process requires the sensitivity to seize the moment in order to determine the materiality of the process further – and to bring out what it solicits most. We thank one of our reviewers for bringing this relation to kairos to our attention. Note that in our figures these moments of alignments are not explicitly represented..
